# Impact of Intraoperative Fluid Balance on Postoperative Clinical Outcomes after Single-Lung Transplantation

**DOI:** 10.5761/atcs.oa.26-00152

**Published:** 2026-10-22

**Authors:** Ken Onodera, Tatsuaki Watanabe, Yusuke Takei, Yui Watanabe, Takaya Suzuki, Takashi Hirama, Hirotsugu Notsuda, Hisashi Oishi, Yutaka Ejima, Yoshinori Okada

**Affiliations:** 1 Department of Thoracic Surgery, Tohoku University Hospital, Sendai, Miyagi, Japan; 2 Department of Anesthesiology, Tohoku University Hospital, Sendai, Miyagi, Japan

**Keywords:** lung transplantation, fluid balance, single-lung transplantation, primary graft dysfunction, multivariable analysis

## Abstract

**Purpose:**

Single-lung transplantation (SLTx) recipients remain vulnerable to intraoperative fluid overload due to the presence of the native diseased lung. This study evaluated the impact of weight-adjusted intraoperative fluid balance on postoperative outcomes in brain-dead donor SLTx recipients.

**Methods:**

We analyzed 88 SLTx recipients categorized into low-fluid (<70 mL/kg; n = 44) and high-fluid (≥70 mL/kg; n = 44) groups based on the median (70.3 mL/kg). Multivariable logistic and linear regression analyses, with natural-log transformation of mechanical ventilation duration and ICU stay, were adjusted for operative duration, blood loss, and extracorporeal life support (ECLS) use.

**Results:**

Donor characteristics were comparable between groups. High fluid balance (≥70 mL/kg) was associated with significantly higher 48-hour primary graft dysfunction (PGD) Grade 3 rates (34.1% vs. 6.8%; P = 0.003), tracheostomy rates (36.4% vs. 9.1%; P = 0.004), longer mechanical ventilation (4 vs. 2 days; P = 0.016), and ICU stay (10 vs. 6 days; P = 0.011). Postoperative ECLS continuation at 48 hours was identical (2.3% vs. 2.3%). In multivariable analysis, high fluid balance remained significantly associated with 48-hour PGD Grade 3 (odds ratio: 4.91; P = 0.039).

**Conclusion:**

A high intraoperative fluid balance was independently associated with severe 48-hour PGD Grade 3 following SLTx, supporting careful attention to intraoperative fluid balance in these patients.

## Abbreviations

CHDF
: continuous hemodiafiltration
CI
: confidence interval
ECLS
: extracorporeal life support
ICU
: intensive care unit
IQR
: interquartile ranges
ISHLT
: International Society for Heart and Lung Transplantation
OR
: odds ratio
P/F
: PaO2/FiO_2_
PGD
: primary graft dysfunction

## Introduction

Lung transplantation is the established definitive therapeutic option for patients with end-stage respiratory failure.^1)^ Despite substantial advances in surgical techniques and perioperative management, postoperative respiratory complications—including primary graft dysfunction (PGD) and prolonged mechanical ventilation—remain major clinical challenges that significantly affect patient recovery and resource utilization.^2)^ Achieving early weaning from mechanical ventilation is a primary goal in the immediate postoperative period, as prolonged ventilation increases the risks of ventilator-associated lung injury, diaphragmatic dysfunction, and nosocomial infections.

Intraoperative fluid management plays a pivotal role in balancing hemodynamic stability with the preservation of the newly implanted pulmonary graft. Because the transplanted lung is highly susceptible to ischemia–reperfusion injury and hydrostatic pulmonary edema resulting from disrupted lymphatic drainage, a restrictive fluid strategy is generally recommended to keep the graft as “dry” as possible.^3,4)^ However, maintaining this restrictive strategy is often particularly difficult during lung transplantation. Massive hemorrhage, prolonged operative duration, and severe hemodynamic instability often necessitate aggressive fluid and blood product resuscitation.^5)^ Consequently, inadvertent intraoperative fluid overload is common, but its independent impact on specific postoperative clinical outcomes remains to be fully elucidated.^6,7)^

A major limitation of previous literature evaluating intraoperative fluid balance is that these studies have combined single and bilateral lung transplantation into a single, heterogeneous cohort.^8)^ Bilateral lung transplantation inherently involves substantially greater surgical trauma, longer operative time, and larger blood loss than single-lung transplantation (SLTx), creating substantial confounding bias that may obscure the true effect of fluid balance. Furthermore, in countries such as Japan, where persistent donor shortages have historically led to a higher proportion of SLTxs, understanding the specific impact of fluid management within each homogeneous surgical population is of particular clinical relevance. To date, few studies have examined the association between the intraoperative fluid balance and postoperative outcomes in SLTx recipients, including PGD grades, the requirement of tracheostomy, the duration of postoperative mechanical ventilation, and the length of intensive care unit (ICU) stay.

Therefore, the purpose of this study was to evaluate the impact of intraoperative fluid balance on postoperative clinical outcomes in recipients of single-lung transplantation from brain-dead donors. Using robust multivariable regression analyses, we aimed to determine whether weight-adjusted intraoperative fluid excess serves as an independent predictor of higher PGD grades, tracheostomy requirements, prolonged mechanical ventilation, and ICU stay, even after controlling for critical intraoperative confounders including operative time, blood loss, and intraoperative extracorporeal life support (ECLS) use.

## Material and Methods

### Patient selection and study design

The study was reviewed and approved by the Institutional Review Board of Tohoku University Hospital (approval no. 2025-1-799) and was conducted in accordance with the Declaration of Helsinki and applicable national regulations. As this analysis used only de-identified registry data, written informed consent was waived; where applicable, participation was disclosed using an opt-out procedure via institutional websites and public notices. All transplants captured in the registries were performed in compliance with Japanese law and internationally accepted ethical standards, including the Declaration of Istanbul and the International Society for Heart and Lung Transplantation (ISHLT) Statement on Transplant Ethics. No organs were procured from executed prisoners or prisoners of conscience.

We retrospectively reviewed the medical records of patients who underwent lung transplantation at Tohoku University Hospital between March 2014 and December 2025. A total of 154 patients were initially identified. To eliminate confounding clinical factors associated with different donor sources, patients who underwent brain-dead donor bilateral-lung transplantation (n = 57) and living-donor lung transplantation (n = 9) were excluded from the analysis. The final primary analysis cohort comprised 88 brain-dead donor SLTx recipients.

The weight-adjusted intraoperative fluid balance up to ICU admission was quantified using the following formula:

$$\begin{array}{l} \text{Weight Adjusted Fluid Balance }\left(\frac{\text{mL}}{\text{kg}}\right)\text{ = }\\ \frac{\text{Total Fluid Intake (mL) }-\text{ Total Fluid Output (mL)}}{\text{Preoperative Body Weight (kg)}} \end{array}$$

where Total Fluid Intake encompassed all intravenously administered fluids (including crystalloids, colloids, and blood products such as red blood cells, fresh frozen plasma, and platelets), and Total Fluid Output included intraoperative blood loss and urine output. The ECLS priming volume did not affect the net fluid balance, as it was balanced out at configuration or decannulation according to our institutional protocol.

Patients were categorized into low-fluid (<70 mL/kg; n = 44) and high-fluid (≥70 mL/kg; n = 44) groups based on the median weight-adjusted fluid balance of the cohort (70.3 mL/kg) (**Fig. 1**).

**Fig. 1:**
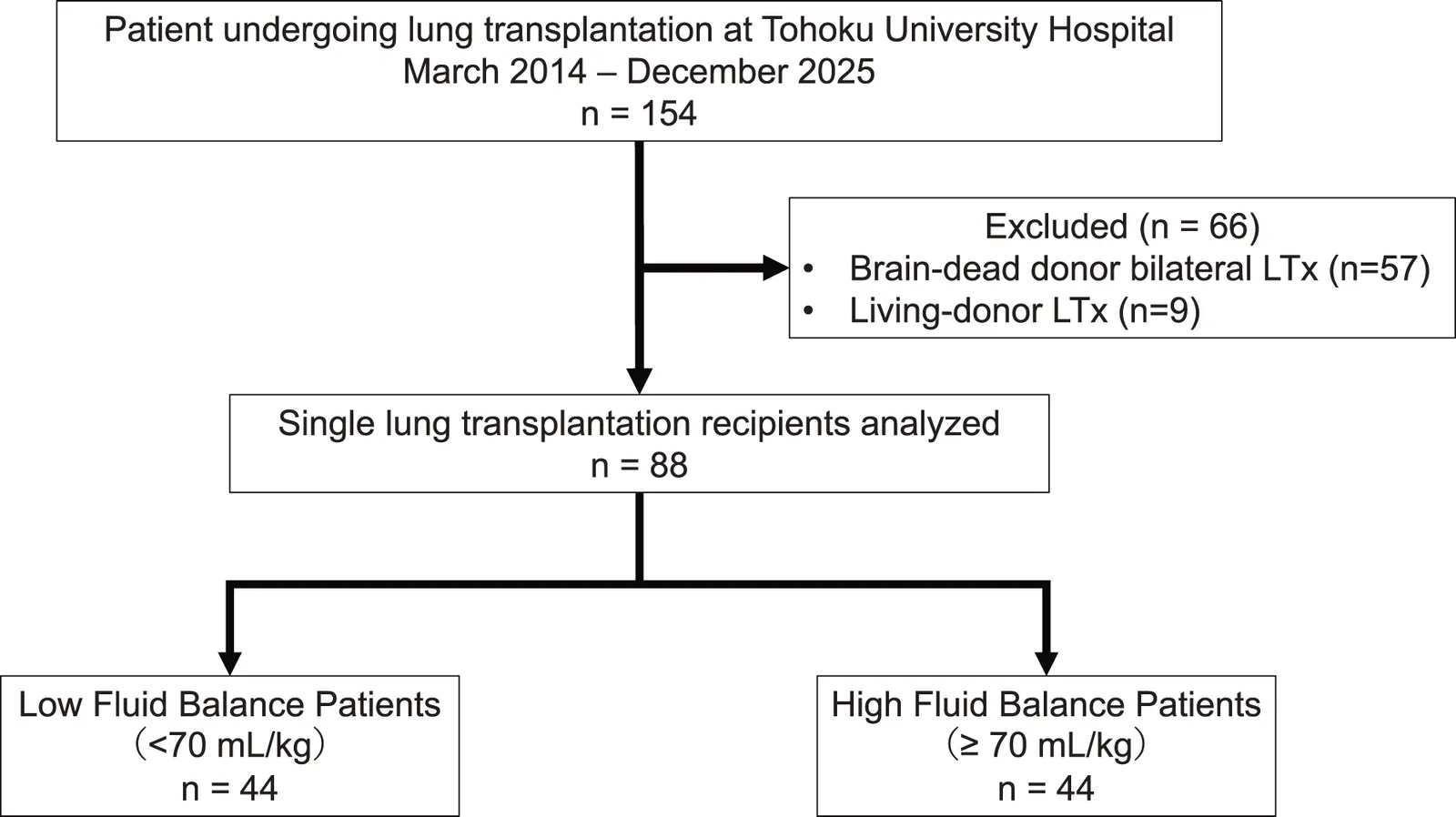
Study flowchart of the patient selection and stratification process. Among the initial 154 patients who underwent lung transplantation, 66 were excluded (57 brain-dead donor bilateral-lung transplantation cases and 9 living-donor lung transplantation cases). The remaining 88 brain-dead donor single-lung transplantation recipients were analyzed and categorized into low-fluid (<70 mL/kg; n = 44) and high-fluid (≥70 mL/kg; n = 44) groups based on the cohort’s median weight-adjusted fluid balance threshold of 70.3 mL/kg. LTx, lung transplantation

### Statistical analysis

Continuous variables are expressed as medians with interquartile ranges (IQRs) and were compared using the Mann–Whitney U test. Categorical variables are presented as counts with percentages and were analyzed using the chi-square test or Fisher’s exact test, as appropriate.

In addition, weight-adjusted intraoperative fluid balance was analyzed as a continuous variable using univariable logistic regression to assess its association with 48-hour PGD Grade 3 without dichotomization.

To evaluate factors associated with adverse postoperative clinical outcomes in SLTx, multivariable regression analyses were performed for the 4 outcomes that demonstrated statistically significant differences in the bivariable analysis: 48-hour PGD Grade 3 as defined by the International Society for Heart and Lung Transplantation,^9)^ tracheostomy requirement, duration of mechanical ventilation, and length of ICU stay. Multivariable nominal logistic regression analyses were utilized for the binary outcomes (48-hour PGD Grade 3 and tracheostomy requirement), whereas multivariable linear regression analyses were performed for the continuous outcomes (duration of mechanical ventilation and ICU stay). Because the distributions of mechanical ventilation duration and ICU stay were right-skewed, these outcomes were natural-log-transformed before multivariable linear regression analysis. For patients who died before liberation from mechanical ventilation or ICU discharge, the respective duration was calculated through the date of death. Multivariable analyses were performed using complete cases; 2 patients with missing intraoperative blood loss data were excluded from these analyses. In all models, variables with clinical relevance or those showing significant imbalances between the fluid groups—specifically operative time, intraoperative blood loss, and intraoperative ECLS use—were forced into the multivariable regression models alongside the high fluid balance indicator (≥70 mL/kg). Intraoperative blood loss was entered into the regression models per 100-mL increase to facilitate interpretation of the effect estimates. All statistical analyses were performed using JMP Student Edition software (version 19.0.4; SAS Institute, Cary, NC, USA). A P-value <0.05 was considered statistically significant.

## Results

### Baseline characteristics and operative parameters of the SLTx cohort

The baseline characteristics and operative parameters of the 88 single-lung transplant recipients are summarized in **Table 1**. The median age was 52 years (IQR, 45–58 years), with a male predominance (61.4%). Fibrosis was the predominant transplant indication (59.1%), followed by obstructive lung diseases (27.3%), graft-vs.-host disease (GVHD) following allogeneic hematopoietic stem cell transplantation (8.0%), and chronic lung allograft dysfunction (CLAD) following previous lung transplantation (5.7%). The median preoperative weight was 51.1 kg (IQR, 42.5–63.3 kg). Regarding intraoperative parameters, the median operative time was 432 min (IQR, 370–480 min), and the median intraoperative blood loss was 855 mL (IQR, 469–1844 mL). The median fluid balance was 3788 mL (IQR, 2707–4928 mL), and intraoperative ECLS was required in 51 cases (58.0%). Following the weight-adjusted stratification based on the median-derived threshold, 44 patients (50.0%) were assigned to the high-fluid group (≥70 mL/kg).

**Table 1:** Baseline characteristics and operative parameters of single-lung transplant recipients

	Single LTx (n = 88)
Baseline characteristics	
Age, years, median (IQR)	52 (45–58)
Gender, male, n (%)	54 (61.4)
Transplant indication, n (%)	
Vascular	0 (0)
Fibrosis	52 (59.1)
Obstructive	24 (27.3)
Suppurative	0 (0)
Allogeneic (GVHD)	7 (8.0)
Allogeneic (CLAD)	5 (5.7)
Operative parameters	
Preoperative weight, kg, median (IQR)	51.1 (42.5–63.3)
Operation time, min, median (IQR)	432 (370–480)
Intraoperative blood loss, mL, median (IQR)	855 (469–1844)
Intraoperative ECLS use, n (%)	51 (58.0)
*Weight Gain, kg, median (IQR)	2.0 (0.8–3.1)
Fluid Balance, mL, median (IQR)	3788 (2707–4928)
High fluid balance patients (≥70 mL/kg), n (%)	44 (50.0)

*Weight gain: the difference between body weight at ICU admission and preoperative body weight.

LTx, lung transplantation; IQR, interquartile ranges; GVHD, graft-vs.-host disease; CLAD, chronic lung allograft dysfunction; ECLS, extracorporeal life support

### Recipient and donor characteristics, operative parameters, and postoperative outcomes

Regarding recipient characteristics, recipient age and transplant side were comparable between the low- and high-fluid groups. Male sex was more frequent in the low-fluid group than in the high-fluid group (75.0% vs. 47.7%; P = 0.015), and recipient BMI was significantly higher in the low-fluid group (median, 22.3 vs. 17.8 kg/m^2^; P <0.001). The distribution of transplant indications did not differ significantly between the groups (P = 0.281). Preoperative bridging was uncommon and did not differ significantly between the groups (P = 0.359). Crucially, there were no significant differences between the 2 groups regarding donor lung quality, including donor age (47 vs. 47 years; P = 0.820), donor smoking history (30.2% vs. 27.3%; P = 0.816), final PaO2/FiO_2_ (P/F) ratio (487 vs. 471 mmHg; P = 0.585), purulent secretions on bronchoscopy (14.0% vs. 9.1%; P = 0.521), infiltrate on chest imaging (30.2% vs. 40.9%; P = 0.372), or the proportion of extended-criteria donors (P = 0.246) (**Table 2**). The high-fluid group exhibited significantly longer operative times (median: 464 vs. 387 min; P <0.001) and higher volumes of intraoperative blood loss (median: 1657 vs. 502 mL; P = 0.0027) than the low-fluid group. Patients in the high-fluid group experienced significantly greater perioperative weight gain (median: 2.5 vs. 1.7 kg; P = 0.0297) and higher total fluid balance (median: 4916 vs. 2833 mL; P <0.001). Furthermore, intraoperative ECLS use was drastically more frequent in the high-fluid group (79.6% vs. 36.4%; P <0.001). Importantly, total ischemic time did not differ significantly between the low-fluid and high-fluid groups (median: 450 vs. 450 min; P = 0.792).

**Table 2:** Recipient and donor characteristics, operative parameters, and postoperative outcomes according to intraoperative fluid balance

	Low-fluid group (<70 mL/kg) (n = 44)	High-fluid group (≥70 mL/kg) (n = 44)	P-value
Recipient characteristics			
Recipient age, years, median (IQR)	55 (45–59)	51 (43–58)	0.320
Recipient sex, male, n (%)	33 (75.0)	21 (47.7)	0.015
Recipient BMI, median (IQR)	22.3 (19.3–24.3)	17.8 (14.8–22.1)	<0.001
Transplant indication, n (%)			0.281
Fibrosis	27 (61.4)	25 (56.8)	
Obstructive	14 (31.8)	10 (22.7)	
Allogeneic (GVHD)	2 (4.6)	5 (11.4)	
Allogeneic (CLAD)	1 (2.3)	4 (9.1)	
Preoperative bridging, n (%)			0.359
Mechanical ventilation	0	1 (2.3)	
ECLS	0	1 (2.3)	
Transplant side, Right, n (%)	24 (54.6)	25 (56.8)	0.830
Donor characteristics			
Donor age, years, median (IQR)	47 (36–56)	47 (38–56)	0.820
Donor smoking, ≥20 pack-years, n (%)	13 (30.2)	12 (27.3)	0.816
Donor P/F ratio, mmHg, median (IQR)	487 (437–523)	471 (417–549)	0.585
Purulent bronchoscopy, n (%)	6 (14.0)	4 (9.1)	0.521
Infiltrate on chest imaging, n (%)	13 (30.2)	18 (40.9)	0.372
Extended donor status			0.246
1 category	11 (25.6)	18 (40.9)	
2 or above	13 (30.2)	13 (30.0)	
Operative parameters			
Operation time, min, median (IQR)	387 (363–442)	464 (392–516)	<0.001
Intraoperative blood loss, mL, median (IQR)	502 (299-943)	1657 (705–3175)	0.003
Intraoperative ECLS use, n (%)	16 (36.4)	35 (79.6)	<0.001
*Weight gain, kg, median (IQR)	1.7 (0.5–2.6)	2.5 (1.5–3.4)	0.030
Fluid balance, mL, median (IQR)	2833 (2120–3613)	4916 (4186–6620)	<0.001
Postoperative outcomes			
Mechanical ventilation, days, median (IQR)	2 (1–4)	4 (1–12)	0.016
Tracheostomy requirement, n (%)	4 (9.1)	16 (36.4)	0.004
ICU stay, days, median (IQR)	6 (4–9)	10 (5–28)	0.011
48-hour PGD Grade 3, n (%)	3 (6.8)	15 (34.1)	0.003
**Post-transplant CHDF requirement, n (%)	2 (4.6)	5 (12.2)	0.255
In-hospital mortality, n (%)	1 (2.3)	2 (4.6)	1.000
30-day mortality, n (%)	0	0	–
90-day mortality, n (%)	0	1 (2.3)	1.000

*Weight gain: the difference between body weight at ICU admission and preoperative body weight.

**CHDF requirements were evaluated for 85 patients (44 in the low-fluid group and 41 in the high-fluid group) due to early mortality or missing records.

IQR, interquartile ranges; BMI, body mass index; GVHD, graft-vs.-host disease; CLAD, chronic lung allograft dysfunction; ECLS, extracorporeal life support; ICU, intensive care unit; PGD, primary graft dysfunction; CHDF, continuous hemodiafiltration; P/F ratio, PaO2/FiO_2_ ratio

Regarding postoperative clinical outcomes, the high-fluid group showed poorer respiratory outcomes in the unadjusted comparisons. Strikingly, severe early graft dysfunction, represented by 48-hour PGD Grade 3, was more than 5-fold higher in the high-fluid group (34.1% vs. 6.8%; P = 0.0029) (**Fig. 2A**). Similarly, the tracheostomy requirement rate was significantly increased in the high-fluid group (36.4% vs. 9.1%; P = 0.0043) (**Fig. 2B**). The median duration of postoperative mechanical ventilation was significantly prolonged in the high-fluid group compared to the low-fluid group (4 vs. 2 days; P = 0.0157). Furthermore, the high-fluid group also demonstrated significantly prolonged ICU stays (median: 10 vs. 6 days; P = 0.0113), while post-transplant continuous hemodiafiltration (CHDF) requirement demonstrated a nonsignificant higher trend in the high-fluid group (12.2% vs. 4.6%; P = 0.2547). Notably, the postoperative ECLS continuation rate at 48 hours was extremely low and identical between the 2 groups (2.3% vs. 2.3%; P >0.999), confirming that the observed increase in PGD Grade 3 was not driven by definition-induced bias related to ongoing ECLS. In-hospital mortality was 2.3% in the low-fluid group and 4.6% in the high-fluid group (P = 1.000), with no 30-day deaths in either group. Ninety-day mortality was 0% and 2.3%, respectively (P = 1.000).

**Fig. 2:**
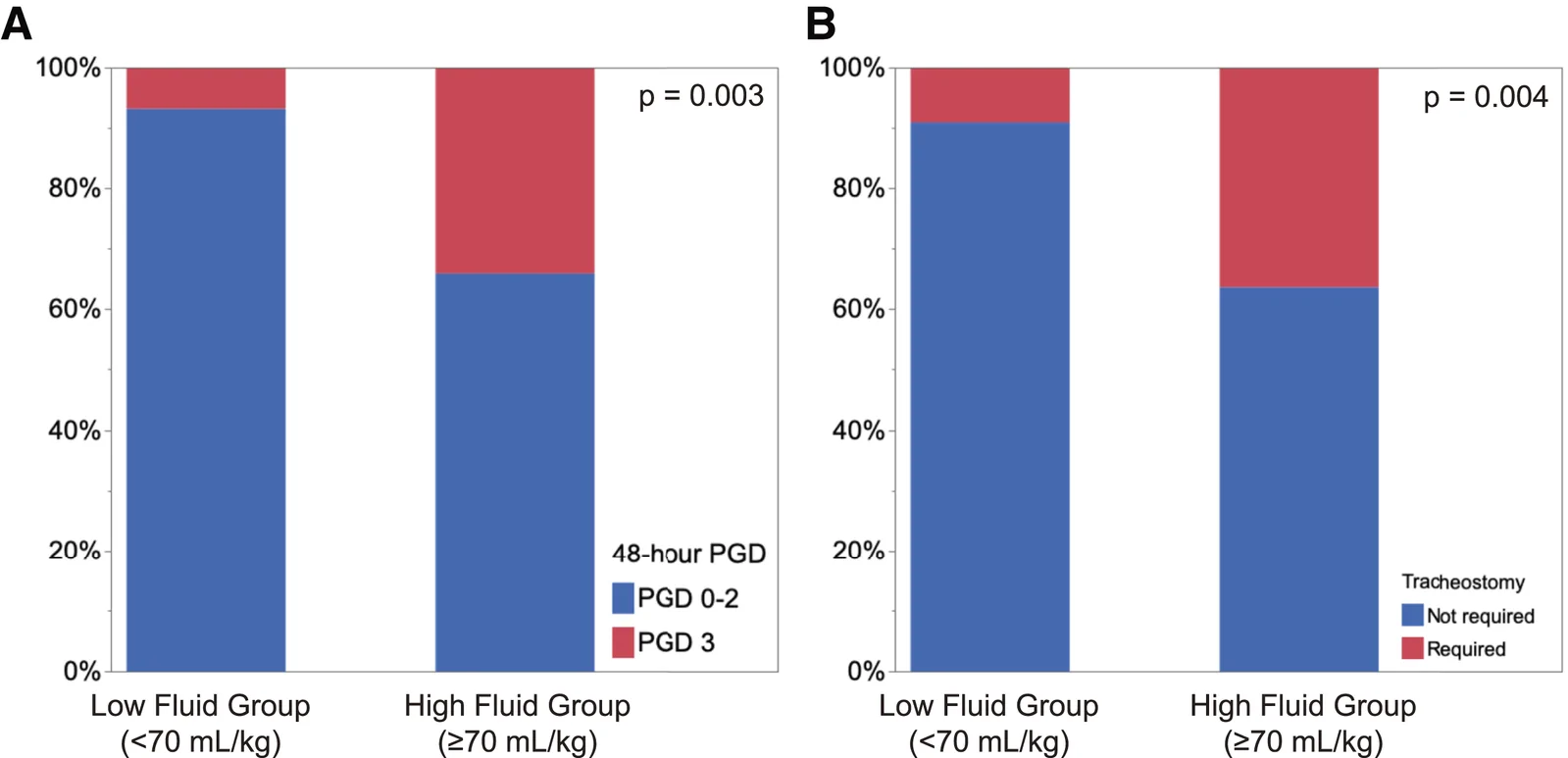
Comparison of postoperative clinical outcomes in single-lung transplantation between the low-fluid group (<70 mL/kg) and high-fluid group (≥70 mL/kg). (**A**) Bar chart comparing the 48-hour PGD Grade 3 rates (%) between the 2 groups. The incidence of 48-hour PGD Grade 3 was significantly higher in the high-fluid group than in the low-fluid group (34.1% vs. 6.8%; P = 0.0029, chi-square test). (**B**) Bar chart comparing the postoperative tracheostomy requirement rates (%) between the 2 groups. The rate of tracheostomy was significantly higher in the high-fluid group than in the low-fluid group (36.4% vs. 9.1%; P = 0.0043, chi-square test). PGD, primary graft dysfunction

### Multivariable analysis for postoperative clinical outcomes

When analyzed as a continuous variable, greater weight-adjusted intraoperative fluid balance was significantly associated with 48-hour PGD Grade 3 in univariable logistic regression analysis (odds ratio [OR] 1.014 per 1-mL/kg increase; 95% confidence interval [CI], 1.005–1.024; P = 0.0033).

To account for the baseline and intraoperative imbalances between the fluid groups, multivariable regression analyses were performed for the 4 postoperative clinical outcomes that demonstrated statistical significance in the bivariable analysis: 48-hour PGD Grade 3, tracheostomy requirement, duration of mechanical ventilation, and duration of ICU stay (**Table 3**).

**Table 3:** Multivariable analysis for postoperative clinical outcomes in single-lung transplantation recipients

	Odds ratio/regression coefficient (β)	95% CI	P-value
Model 1: 48-hour PGD grade 3 (Odds ratio)			
High fluid balance patients (≥70 mL/kg)	4.906	1.080–35.017	0.039
Operation time	1.002	0.996–1.008	0.489
Intraoperative blood loss (per 100 mL)	1.006	0.991–1.027	0.433
Intraoperative ECLS use	6.493	0.989–128.547	0.052
Model 2: Tracheostomy requirement (Odds ratio)			
High fluid balance patients (≥70 mL/kg)	3.439	0.802–14.741	0.096
Operation time	0.998	0.992–1.005	0.578
Intraoperative blood loss (per 100 mL)	0.982	0.948–1.002	0.174
Intraoperative ECLS use	2.992	0.532–16.816	0.214
*Model 3: Mechanical ventilation (Regression coefficient β)			
High fluid balance patients (≥70 mL/kg)	0.061	−0.200–0.322	0.643
Operation time	0.004	0.002–0.007	0.002
Intraoperative blood loss (per 100 mL)	0.009	0.001–0.017	0.020
Intraoperative ECLS use	0.325	−0.225–0.875	0.244
*Model 4: ICU stay (Regression coefficient β)			
High fluid balance patients (≥70 mL/kg)	0.056	−0.111–0.223	0.504
Operation time	0.002	0.000–0.004	0.017
Intraoperative blood loss (per 100 mL)	0.009	0.004–0.014	0.001
Intraoperative ECLS use	0.254	−0.098–0.605	0.155

*Mechanical ventilation duration and ICU stay were natural-log-transformed for multivariable linear regression analyses.

CI, confidence interval; ECLS, extracorporeal life support; ICU, intensive care unit; PGD, primary graft dysfunction

For the binary outcomes, multivariable nominal logistic regression analyses were utilized. A high intraoperative fluid balance (≥70 mL/kg) remained a powerful, independent predictor of severe early graft injury, conferring a nearly 5-fold increase in the odds of 48-hour PGD Grade 3 (OR: 4.91; 95% CI, 1.08–35.02; P = 0.0387) (Model 1). Intraoperative ECLS use also demonstrated a strong trend toward 48-hour PGD Grade 3 (OR: 6.49; 95% CI, 0.99–128.55; P = 0.0516), whereas operative time (P = 0.4891) and intraoperative blood loss (P = 0.4328) lost their statistical significance. Conversely, in Model 2 (predicting tracheostomy requirement), the independent association of high fluid balance was attenuated after adjusting for operative duration and ECLS use (OR: 3.44; 95% CI, 0.80–14.74; P = 0.0963). No other single intraoperative factor, including ECLS use (P = 0.2135), operative time (P = 0.5780), or blood loss (P = 0.1735), served as an independent statistical predictor for tracheostomy requirement.

For the continuous outcomes, multivariable linear regression analyses were performed. A high intraoperative fluid balance (≥70 mL/kg) showed no independent association with prolonged ventilation (Regression Coefficient [β] = 0.061, P = 0.643) (Model 3). Instead, longer operative time (β = 0.004, P = 0.002) and greater intraoperative blood loss (β = 0. 0093, P = 0.020) were independently associated with prolonged mechanical ventilation. Similarly, in Model 4, a high fluid balance was not independently associated with the duration of ICU stay (β = 0.056, P = 0.504). Longer operative time (β = 0.002, P = 0.017) and greater intraoperative blood loss (β = 0.0086, P = 0.001) were independently associated with longer ICU stay, while intraoperative ECLS use was not statistically significant (β = 0.254, P = 0.155).

## Discussion

The primary finding of the present study is that a high intraoperative fluid balance (≥70 mL/kg) was significantly associated with severe early graft injury (48-hour PGD Grade 3) in patients undergoing SLTx from brain-dead donors. Multivariable logistic regression analysis showed that the association remained significant after adjustment for operative time, blood loss, and intraoperative ECLS use. However, these intraoperative factors are closely interrelated, and statistical adjustment cannot fully distinguish the effect of fluid balance from that of surgical complexity and hemodynamic instability.^6,7)^ Moreover, greater blood loss and ECLS use may both increase fluid requirements and reflect intraoperative instability; therefore, a high positive fluid balance may partly represent a marker of intraoperative complexity or instability rather than a direct cause of PGD. Importantly, donor lung characteristics, including donor P/F ratio, smoking history, chest imaging abnormalities, and extended-criteria donor status, were comparable between the 2 groups, suggesting that the observed association was unlikely to be explained solely by differences in measured donor lung quality.

Our findings suggest that a high intraoperative fluid balance is particularly associated with acute graft dysfunction (48-hour PGD Grade 3), whereas its association with tracheostomy requirement, duration of mechanical ventilation, and ICU stay was no longer statistically significant after multivariable adjustment. This difference deserves careful pathophysiological consideration. A newly implanted lung graft is highly vulnerable to volume overload for several anatomical and physiological reasons. First, during surgery, the lymphatic drainage network is completely disrupted.^10)^ Therefore, the transplanted lung cannot clear excess fluid on its own. Second, ischemia–reperfusion injury increases vascular permeability, causing fluid to move into the alveolar and interstitial spaces even under normal pressures. Furthermore, in SLTx, because the diseased native lung often exhibits high vascular resistance, a major portion of cardiac output is preferentially directed into the compliant, newly revascularized allograft. Excess positive fluid balance may exacerbate alveolar–capillary leakage in this single graft, precipitating acute pulmonary edema. In contrast, later outcomes, such as the need for a tracheostomy, mechanical ventilation days, and the duration of ICU stay, were not determined by fluid balance alone. This suggests that although excessive positive fluid balance may contribute to acute graft injury, long-term recovery and hospital resource use are multifactorial outcomes driven by a combination of surgical stress, inflammation, and baseline patient condition.^11,12)^

Another strength of this study is our stratified approach. By analyzing SLTx separately, we could better delineate the independent impact of fluid management. Most previous studies combined single- and bilateral-lung transplantations into a single cohort. However, bilateral-lung transplantation inherently requires much longer operative times and results in greater blood loss. This often necessitates aggressive fluid resuscitation, placing almost all bilateral patients in the high-fluid group. In such mixed cohorts, fluid balance simply reflects surgical complexity, creating strong confounding. By isolating the single-lung transplant cohort, which represents a relatively homogeneous surgical model, we minimized these major confounding factors, allowing us to more specifically evaluate the association between intraoperative fluid balance and postoperative pulmonary outcomes. Because the severe donor shortage in Japan historically leads to a higher rate of SLTx compared to Western registries, our findings have important clinical implications for improving patient outcomes in this specific population.

From a clinical standpoint, our data raise caution regarding the liberal administration of fluid to manage hemodynamic instability or bleeding during surgery. While maintaining adequate organ perfusion is essential, clinicians must recognize that fluid resuscitation during lung transplantation should not be considered benign. The strong independent association between a fluid balance ≥70 mL/kg and 48-hour PGD Grade 3 suggests that fluid management requires careful consideration. Early and proactive use of vasopressors or inotropes to maintain systemic perfusion, rather than relying solely on fluid boluses, should be considered to keep the graft dry and avoid the severe acute respiratory complications found in this study. Importantly, a restrictive fluid management strategy must not be misinterpreted as simple fluid deprivation; rather, it should be predicated on a meticulous hemodynamic strategy in which appropriate vasoactive agents, such as norepinephrine or vasopressin, are optimized by anesthesiologists to sustain systemic perfusion while keeping the pulmonary graft dry. Furthermore, the postoperative ECLS continuation rate at 48 hours was extremely low and identical between the 2 groups (2.3% vs. 2.3%; P >0.999), confirming that the observed increase in severe early graft injury was driven by primary pulmonary parenchymal dysfunction rather than definition-induced bias related to ongoing ECLS. In our cohort, CHDF requirement was not significantly associated with intraoperative fluid overload. In contrast, Shen et al. recently reported a significant association between fluid overload and postoperative acute kidney injury following lung transplantation.^13)^ The reasons for this discrepancy remain unclear and warrant further investigation.

Several limitations of this study should be noted. First, this was a retrospective, single-center study, which introduces selection bias and limits the generalizability of our findings. In addition, the possibility of residual confounding and reverse causation cannot be excluded. Patients with greater surgical complexity, bleeding, or hemodynamic instability may have required greater fluid and blood product administration, and therefore a high positive fluid balance may partly represent a marker of intraoperative instability rather than a direct cause of PGD. Although operative time, blood loss, and ECLS use were included in the multivariable analysis, these factors are closely interrelated with fluid balance. In particular, blood loss is incorporated into the calculation of net fluid balance, while bleeding itself leads to fluid and blood product administration, and ECLS use may both reflect intraoperative instability and influence fluid requirements. Thus, blood loss and ECLS use may act not only as confounding factors but also as intermediate variables within the causal pathway, and statistical adjustment cannot fully disentangle these relationships. Second, the sample size of our primary analysis cohort was relatively small, although it represents one of the largest single-center cohorts of SLTx evaluated for fluid balance. Third, the 70-mL/kg cutoff was derived from the median fluid balance of the present cohort and should not be interpreted as a clinically validated therapeutic threshold. Although the association with 48-hour PGD Grade 3 was also observed when fluid balance was analyzed as a continuous variable, potential nonlinear or threshold effects could not be adequately evaluated in this relatively small cohort and should be investigated in larger multicenter studies. Fourth, detailed data regarding the specific types of intraoperative fluids, such as the ratio of crystalloids to colloids, the amounts of individual blood products, and early postoperative ventilator settings, were not fully evaluated. Fifth, due to the retrospective design, detailed data on the doses and timing of intraoperative vasopressors were unavailable. Finally, for patients returning to the ICU with ongoing ECLS, the precise fluid shifts related to the priming volume could not be fully evaluated.

## Conclusions

In conclusion, a high intraoperative fluid balance (≥70 mL/kg) was independently associated with 48-hour PGD Grade 3 in patients undergoing single-lung transplantation from brain-dead donors. This independent relationship remained significant even after adjustment for operative time, intraoperative blood loss, and intraoperative ECLS use, and was independent of preexisting donor lung quality. Careful intraoperative volume monitoring and a restrictive fluid strategy during lung transplantation may help protect the vulnerable lung graft from severe early graft dysfunction.

## Declarations

### Availability of data and materials

The datasets used and analyzed during the current study are available from the corresponding author on reasonable request.
